# Age at Tumor Diagnosis in 14,636 Canine Cases from the Pathology-Based UNIPI Animal Cancer Registry, Italy: One Size Doesn’t Fit All

**DOI:** 10.3390/vetsci11100485

**Published:** 2024-10-08

**Authors:** Niccolò Fonti, Francesca Parisi, Alessio Lachi, Elena Sophie Dhein, Franco Guscetti, Alessandro Poli, Francesca Millanta

**Affiliations:** 1 Department of Veterinary Sciences, University of Pisa, Viale delle Piagge n. 2, 56124 Pisa, Italy; francesca.parisi@unipi.it (F.P.); alessandro.poli@unipi.it (A.P.); francesca.millanta@unipi.it (F.M.); 2 Saint Camillus International University of Health and Medical Sciences (UniCamillus), Via Sant’Alessandro n. 8, 00131 Rome, Italy; alessio.lachi@unicamillus.org; 3 Department of Statistics, Computer Science, Applications “Giuseppe Parenti” (DiSIA), University of Florence, Viale Giovanni Battista Morgagni 59, 50134 Florence, Italy; 4 Institute of Veterinary Pathology, Vetsuisse Faculty, University of Zurich, Winterthurerstrasse 268, 8057 Zurich, Switzerland; elena.dhein@uzh.ch (E.S.D.); franco.guscetti@vetpath.uzh.ch (F.G.)

**Keywords:** age, neoplasia, dogs, Animal Cancer Registry, veterinary oncology, sex, neutering status, breed, cancer screening, cephalic index

## Abstract

**Simple Summary:**

Cancer is the leading cause of death in adult dogs. All dogs would benefit from early diagnosis, but there are no official guidelines for cancer screening in pets, although many different diagnostic tools for cancer detection are being studied. With our analysis of 14,636 cases, we aimed to determine the age of cancer diagnosis for dogs. Malignant tumors were diagnosed more often than benign neoplasms in older dogs. Intact females, large-sized, brachy-/dolichocephalic, and purebred dogs developed cancer at a younger age, but wide differences between breeds were noted. The earliest age at diagnosis was recorded for lymphomas and mast cell tumors. Due to the wide phenotypic and genetic variability of canines, breed-based screening schedules should be devised.

**Abstract:**

Cancer is the most common cause of death in adult dogs. All dogs would benefit from early diagnosis, but there are no specific guidelines regarding the schedule of cancer screening in companion animals. The aim of this study was to retrospectively evaluate the age at diagnosis in Italian oncological canine patients. A total of 14,636 canine histologically confirmed neoplastic cases were coded according to the Vet-ICD-O-canine-1 and stratified by malignancy, sex, neutering status, breed, cephalic index, body size, and tumor type. Differences in age distribution were analyzed and the influence of these variables on the time of first malignancy diagnosis was assessed using an event history analysis model. The median age at diagnosis for benign and malignant tumors was 9 and 10 years, respectively. Intact and purebred dogs were diagnosed earlier, but the median age differed significantly by breed. The earliest age at diagnosis was recorded for lymphomas and mast cell tumors. The model showed an accelerating effect of large size, brachy- and dolichocephaly, and sexual integrity in female dogs on the time of malignancy diagnosis. Our results confirm that a “one-size-fits-all” approach to cancer screening is not accurate in dogs and provide relevant data that may lead to the establishment of breed-based screening schedules.

## 1. Introduction

Cancer represents a major threat to global public health [1]. In recent years, the incidence of neoplastic diseases has been on the rise, with millions of new cases diagnosed annually in both humans and non-human animals [2,3,4]. The life expectancy of companion pet dogs in developed countries has increased significantly since the late 20th century, when dogs started to be increasingly introduced into homes as family members [5]. This increase in life expectancy, and therefore age-related diseases, is also related to the need of many owners to provide their pets with the same level of care as they provide themselves [6]. From a *One Health* perspective, canine cancer data can also provide an innovative and unique animal model for the study of cancer, with several canine malignancies closely resembling their human counterparts [2,7,8].

Neoplastic diseases are more multifaceted in dogs than in humans due to the wide phenotypic and genetic variability among individuals of different breeds [9,10,11,12]. Dog breeds vary in their predisposition to certain malignancies, but all adult dogs share cancer as the leading cause of death, accounting for around 15–30% of all deaths [13,14,15].

Therefore, all dogs would benefit from routine preventive screening, as early detection of cancer is essential to improve the prognosis of many malignancies. Consequently, we are witnessing a growing body of research related to blood-based biopsies and other non-invasive tests [16]. These novel diagnostic techniques offer promising tools for the early detection of cancer, complementary to the conventional methods currently used in cancer screening [16,17,18]. However, an important question is the appropriate age at which to begin cancer screening in dogs, and how endogenous and environmental factors may influence this timepoint.

Starting from the 1960s, several sources have been used to provide data on cancer occurrence, such as insurance databases, owner questionnaires, clinical records, and pathology- or population-based animal cancer registries (ACRs) [19]. More recently, many scientists are working on the implementation of veterinary cancer registries, with the emergence of organizations such as the Global Initiative for Veterinary Cancer Surveillance (GIVCS), which aims to standardize and guide current and future veterinary cancer registries worldwide [20]. To achieve a quality of healthcare in veterinary oncology truly comparable to that in humans, greater emphasis should be placed on the standardization of the collection, monitoring, and sharing of veterinary cancer data [21,22]. This is also true for Italy, where spatially and temporally limited ACRs have been operating since the 1980s [23,24,25,26,27]. Currently, the Italian Network of Laboratories for Veterinary Oncology (NILOV), to which the pathology-based UNIPI ACR has been affiliated since 2019, collects pet tumor diagnoses from various sources, including public veterinary laboratories, universities, and private laboratories, in a single database [28].

Several investigations have focused on tumor incidence rates (IRs) [23,24,25,26,29,30,31,32,33,34,35] or other effect size measures of cancer development such as odds ratios (ORs) [36,37,38,39,40], proportional morbidity ratios (PMRs) [28], or standardized morbidity ratios (SMRs) [41]. However, few studies have aimed to evaluate the variation in age at tumor diagnosis between canine sub-populations [42] and, unlike in human medicine, there are no standardized official guidelines for the optimal time to begin cancer screening in veterinary medicine [43,44].

The aim of this study was to retrospectively assess the age at tumor diagnosis according to sex, breed, size, morphological features, and most common cancer types in a large sample of Vet-ICD-O-canine-1-coded Italian oncological canine patients to provide a basis for effective cancer screening in dogs.

## 2. Materials and Methods

### 2.1. Data Source

The study was designed as a cross-sectional study based on canine tumor diagnoses collected from the pathology-based Animal Cancer Registry (ACR) of the University of Pisa, Italy. All data were obtained from routine histological examinations of formalin-fixed tissue samples, performed in a diagnostic histopathology laboratory between January 2008 and December 2023. Submitting clinicians provided animal owners’ informed consent to protect their privacy and to allow the use of anonymized data in research projects. When necessary, special and immunohistochemical staining were performed to make a definitive diagnosis. Each diagnostic record represented one tumor and included the following information: species, breed, age, sex, neutering status, postal code area, individual ID, symptoms (including anamnestic information and tumor staging), histologic description, diagnosis, and anatomic body localization of the tumor. Inclusion of the tumor cases was not limited by the presence of one or more pieces of missing information provided by the clinician. Each record was subjected to a rigorous screening process to ensure its accuracy, and only cases with definitive, confirmed diagnoses were included. Only primary tumors were included in the analysis. Recurrences (diagnoses of the same tumor in the same animal at a later time) were excluded, but the age at first tumor diagnosis was maintained. In the case of multiple tumors (i.e., different tumors with different histotypes in the same dog), diagnoses were processed according to WHO ICD-O-3.1 guidelines [35,45].

### 2.2. Data Subsetting

Tumor histotype (morphology) and anatomical location (topography) were coded according to the Vet-ICD-O-canine-1 coding system [22]. Diagnoses were divided by a dichotomous variable called “malignancy” (benign “B” vs. malignant “M”) based on the behavioral code. Tumors coded with *[/0]* were classified as benign and *[/3]* as malignant. In the case of “*uncertain whether benign or malignant” [/1]* and “in situ” [/2] neoplasms, malignancy was assigned according to the scheme proposed by Dhein et al. [35]. The only exceptions were *cutaneous mast cell tumor [9740]*, which was considered malignant regardless of grade [39,46,47], and in situ carcinoma arising in the *mammary gland [C50]*, for which there are no standardized criteria to distinguish it from epitheliosis [48], and that was therefore excluded. Malignant tumors were further subdivided by histotype, and the 10 most common histotypes obtained from the Vet-ICD-O-canine-1 morphological “group” levels were analyzed.

Dog breed names were cleaned to remove typographical errors and classified according to the Fédération Cynologique Internationale (FCI) [49]. Unspecified phylogenetically close breeds were grouped as “not otherwise specified” (NOS), as previously described [12,35,50]. All confirmed breeds were included in the “purebred” group for comparison with “mixed-breed” dogs. In addition, the age at tumor diagnosis was calculated for specific breeds with ≥50 cases in the dataset.

For the purebred dogs included in the dataset, body size classification into “small”, “medium”, or “large”, and cephalic index (i.e., average ratio of skull width to length) into “brachycephalic”, “mesocephalic”, or “dolichocephalic” were determined according to McMillan et al. [12,51]. In adherence to previous studies, such classification was not applied to mixed-breed dogs due to their high phenotypic variation [12].

### 2.3. Statistical Analysis

#### 2.3.1. Univariate Analysis

Data cleaning and statistical analysis were performed using Microsoft Excel version 16.84 (Microsoft 2024, www.microsoft.com, accessed on 3 September 2024) and R Language and Environment for Statistical Computing studio R version 4.4.0 [52]. Data visualization was performed using the ggplot2 package (v3.3.3; Wickham, 2016) [53].

Mean and median age at tumor diagnosis were determined for the entire sample and for each subset by malignancy, sex, neutering status, size, cephalic index, breed, and histotype. Age was considered as a continuous variable, and normality was assessed graphically and by the Shapiro–Wilk test. Since data were not normally distributed, differences in age distribution were assessed using non-parametric tests. Mann–Whitney U and Kruskal–Wallis tests were performed with two or more groups, respectively. Dunn’s test with Benjamini–Hochberg *p*-value adjustment was used for post hoc analysis, if the Kruskal–Wallis test was significant. Results with *p* < 0.05 were considered statistically significant.

#### 2.3.2. Event History Analysis

Event history analysis (EHA) was performed on dogs for which all information on sex, castration status, size and cephalic index was reported. EHA allows the study of patterns and correlations according to which a given event occurs. The event is defined as a qualitative change of the unit of analysis, from a state *j* (non-diagnosed) to a state *k* (diagnosed) that occurs at a given time. This setting allows the investigation of not only the type of change, but also when it occurs, since the event requires a preceding time interval that represents its non-occurrence [54]. The key statistical concept of EHA is the transition (or hazard) rate, which expresses the instantaneous risk that the event will occur at time *t’*, given that the event did not occur before time *t*. In EHA, the transition rate is the response variable that depends on a set of independent covariates and time. In this way, EHA is able to analyze the processes that lead to the observed outcome.

The EHA is based on the individual. Thus, we approached the data differently than univariate statistics, in which the age at diagnosis of primary tumors was investigated, regardless of whether they were multiple tumors in a single individual.

As the available dataset pertains to tumor-diagnosed dogs (malignant and benign), we used an EHA model that included the malignant tumor as the negative outcome (event 1), and the benign tumor diagnosis as the positive outcome (event 0). A reduced subset of the entire dataset was therefore subjected to EHA, as multiple tumor diagnoses in the same individual were omitted, and only the biological behavior (malignant or benign) of the tumor firstly diagnosed was retained. If both malignant and benign diagnoses were given at the same time to one individual, only the former was retained. Consequently, the calculated survival and hazard functions refer to the time interval before the diagnosis of a tumor occurs (outcome). Males, neutered dogs, small-sized dogs, and mesocephalic breeds were defined as references for the respective independent covariates. This analysis was performed using STATA18.

## 3. Results

### 3.1. Demographic Features of the Study Population

During the study period of January 2008—December 2023, a total of 17,123 histologic diagnoses of neoplasia were retrieved from the pathology-based UNIPI ACR. After removing 2487 strings—including metastases, recurrences, and cases where the age of the subject was not reported—14,636 cases from 13,189 dogs were included in the study. A summary of the sample size by sex, castration status, breed, size, and cephalic index is presented in Table 1.

Females and males were equally represented, and most cases (10,772; 73.6%) were from intact animals. This tendency was more pronounced in the male group. More than half (8668; 59.2%) of the cases were classified as malignant, while 5968 records were benign (40.8%).

The three most common primary tumor locations were skin [C44] (34.5%), mammary gland [C50] (23.9%), and soft tissue [C49] (12.6%).

Regarding tumor types, *adenomas and adenocarcinomas [814–838]* were the most frequently reported neoplastic morphology group, followed by *complex mixed and stromal neoplasms [893–899]*, *mast cell neoplasms [974]*, *adnexal and skin appendage neoplasms [839–842]*, and *blood vessel tumors [912–916].* The number of cases included in the complete dataset for each tumor topography and morphology are described in Table S1.

### 3.2. Age at Tumor Diagnosis

**Figure 1:**
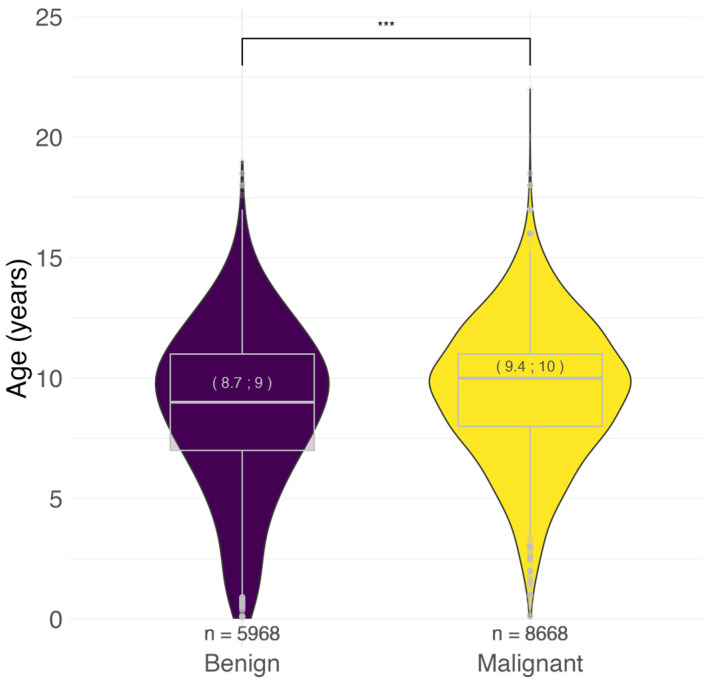
Age distribution of benign and malignant tumors. In brackets: mean; median age at diagnosis in years. *** *p* < 0.001, Mann–Whitney U test. n = number.

Considering the entire dataset, the age at tumor diagnosis ranged from <1 to 22 years. The mean and median ages were lower for benign tumors (8.7 and 9.0 years, respectively) than for malignant tumors (9.4 and 10.0 years) (Figure 1).

Because the focus of our study was to examine the age of onset of malignancies due to their impact on canine health, we limited the following analysis to malignant tumors.

#### 3.2.1. Age at Malignant Tumor Diagnosis by Sex and Neutering Status

**Figure 2:**
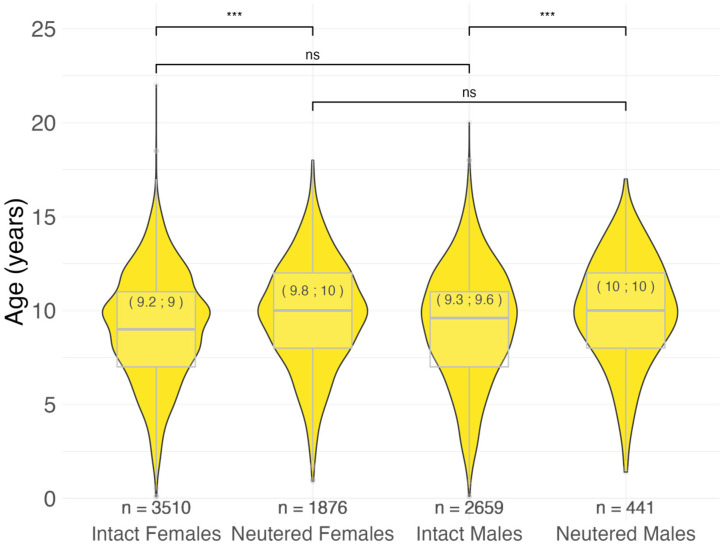
Age at malignant tumor diagnosis by sex and neutering status in 8486 cases. In brackets: mean; median age at diagnosis in years. *** *p* < 0.001, ns = not significant (*p* > 0.05), Pairwise Wilcoxon rank sum test. n = number.

Intact females showed a significantly younger age at malignant tumor diagnosis compared to their neutered counterparts (9.0 vs. 10.0; *p* < 0.001). The same result was observed in males (9.6 vs. 10.0; *p* < 0.001). There was no statistically significant difference between the two sexes in intact (9.0 vs. 9.6; *p* = 0.16) and neutered (10.0 vs. 10.0; *p* = 0.47) dogs (Figure 2).

#### 3.2.2. Age at Malignant Tumor Diagnosis by Size and Cephalic Index

The age at malignant tumor diagnosis was largely influenced by size (*p* < 0.001) and cephalic index (*p* < 0.001). Large dogs had an earlier age at the time of diagnosis (mean 8.6; median 9.0 years) than medium (mean 9.3; median 10.0 years) and small dogs (mean 9.5; median 10.0 years). In contrast, there was no significant difference between small and medium dogs (*p* = 0.16). Brachycephalic dogs were diagnosed with malignancy at a younger age than mesocephalic dogs (median 8.0 vs. 9.0 years, respectively, *p* < 0.001). Dolichocephalic dogs also showed a statistically significant earlier age at diagnosis than mesocephalic dogs (*p* = 0.002), although the two groups had the same median age (9.0 vs. 9.0 years).

#### 3.2.3. Age at Malignant Tumor Diagnosis by Breed

Purebred dogs had a significantly (*p* < 0.001) younger age at malignant tumor diagnosis (mean 8.9; median 9.0 years) compared to mixed-breeds (mean 10.1; median 10.0 years). The median age at malignant tumor diagnosis for the distinct breeds included in the 9333 purebred dogs in our study ranged from 2.6 (American Bully) to 15 years of age (Bobtail). Table S2 presents the size and cephalic index classification, the number of cases, the range, the mean, and the median age at diagnosis for all the 156 breeds in the dataset.

**Figure 3:**
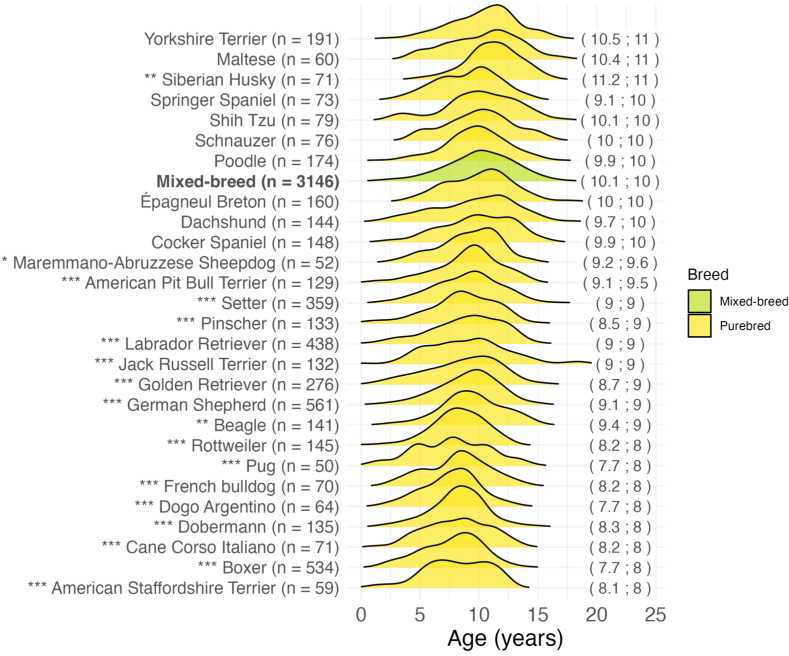
Age at malignant tumor diagnosis for the most common breeds (>50 cases) in 7671 cases. In brackets on the right: mean; median age at diagnosis in years. * *p* < 0.05, ** *p* < 0.01, *** *p* < 0.001, Dunn’s test for “purebred” vs. “mixed-breed” comparison. n = number.

For the breeds represented with more than 50 cases, the age distribution was compared with the mixed-breed group. The results are presented in Figure 3. The American Staffordshire Terrier, Boxer, Italian Cane Corso, Dobermann, Dogo Argentino, French Bulldog, Pug, and Rottweiler breeds showed the youngest median age at malignant tumor diagnosis (8.0 years) and a significantly lower age compared to the mixed-breed group (*p* < 0.001). Other early diagnosed breeds were Beagle (*p* = 0.002), German Shepherd (*p* < 0.001), Golden Retriever (*p* < 0.001), Jack Russell Terrier (*p* < 0.001), Labrador Retriever (*p* < 0.001), Pinscher (*p* < 0.001), and Setter (*p* < 0.001) with a median age of 9.0 years. The highest median age (11.0 years) was observed in the Yorkshire Terrier (*p* = 0.14), Maltese (*p* = 0.52), and Siberian Husky (*p* = 0.002).

#### 3.2.4. Age at Malignant Tumor Diagnosis by Histotype

**Figure 4:**
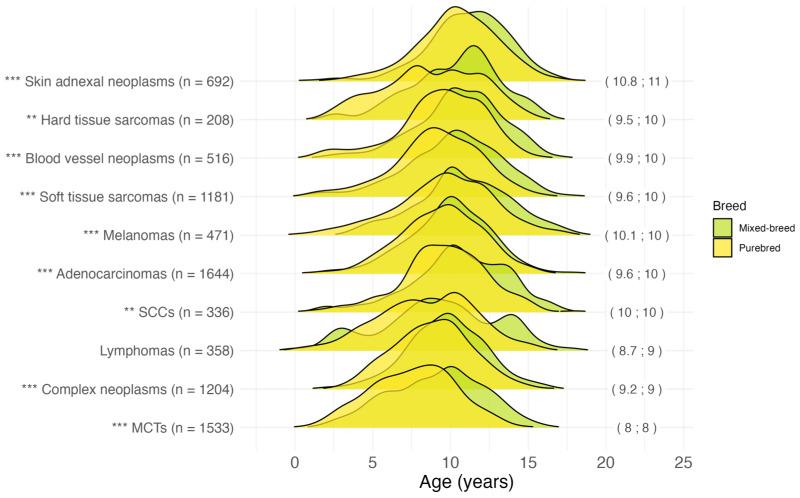
Age at tumor diagnosis for the 10 most common malignant histotypes in 8143 cases. In brackets on the right: mean; median age at diagnosis in years for the overall dog population (mixed-breed and purebred). The following Vet-ICD-O-canine-1 codes were used for histotype classification: “Skin adnexal neoplasms” = [839–842], “Hard tissue sarcomas” = [918–924], “Blood vessel neoplasms” = [912–916], “Soft tissue sarcomas” = [880–892, 954–957], “Melanomas” = [972–892], “Adenocarcinomas” = [814–838], “SCCs” (Squamous cell carcinomas) = [805–808], “Lymphomas” = [959–972], “Complex neoplasms” = [893–899], “MCTs” (Mast cell tumors) = [974]. ** *p* < 0.01, *** *p* < 0.001, Pairwise Wilcoxon rank sum test for “purebred” vs. “mixed-breed” comparison for each histotype. n = number.

The age at diagnosis for the most common malignant tumor histotypes, grouped by breed, is shown in Figure 4. The earliest age at diagnosis was recorded for *mast cell neoplasms [974]* (mean 8.0; median 8.0 years), and *lymphomas [959–972]* (mean 8.7; median 9.0 years). Malignant *adnexal and skin appendage neoplasms [out of 839–842]*, *squamous cell carcinomas [out of 807–808]*, *melanomas [out of 872–879]*, and malignant *blood vessel tumors [912–916]* were diagnosed in the oldest patients, with an overall median age at diagnosis of over 10 years. For all histotypes except lymphoma (*p* = 0.08), purebred dogs were diagnosed at a younger age than mixed-breed dogs.

### 3.3. Event History Analysis

**Figure 5:**
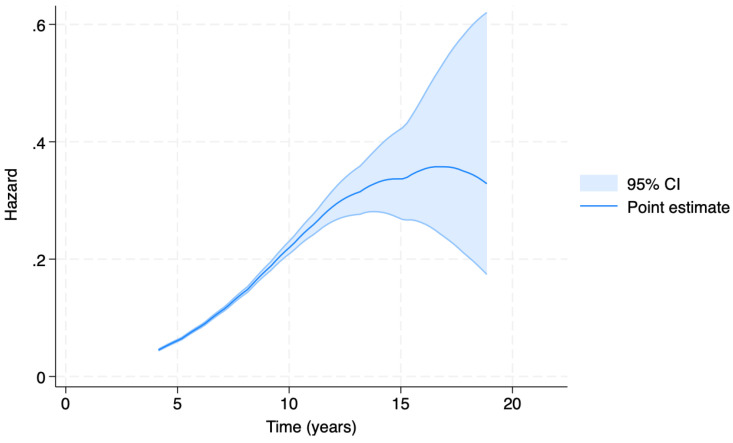
Accelerated Failure Time (AFT) model-generated estimated hazard function. The hazard rate (*y* axis) expresses the instantaneous risk that the event of malignant tumor diagnosis occurs at time *t’*, given that the event did not occur before time *t* (*x* axis).

Of the 13,181 dogs included in the study, complete information on the covariates included in the model was available for 8346 purebred dogs. Given the shape of the hazard function (log-logistic function) reported in Figure 5, we performed a log-logistic EHA model using the Accelerated Failure Time (AFT) parameterization. This parameterization allows us to interpret the effects of the model by assuming that the effect of a covariate is to accelerate or delay the occurrence of the event. For example, if the time scale is measured in years and a continuous variable has a coefficient of 0.5 (−0.5), this indicates that an increase (decrease) of one unit in this variable corresponds to an increase (decrease) of half a year in survival time.

The results of the AFT model are shown in Figure 5. The estimated hazard function shows the highest value between 13 and 17 years of age. The 95% confidence interval then widens due to the small number of geriatric individuals who received their first tumor diagnosis after the age of 15.

**Figure 6:**
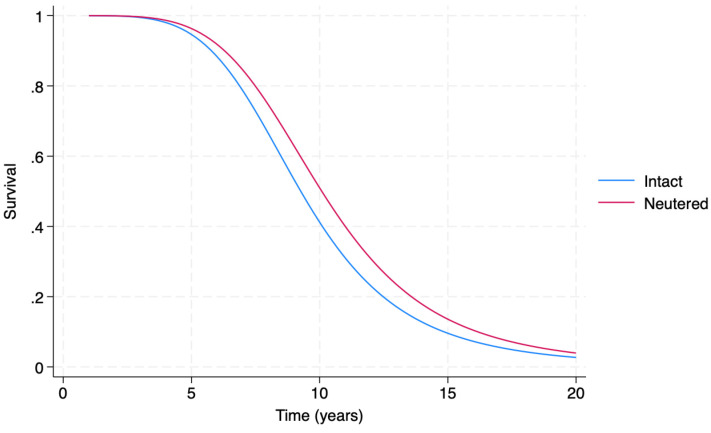
Accelerated Failure Time (AFT) model-generated estimated survival functions in respect to malignant tumor diagnosis in female dogs, providing a comparison of intact (blue line) vs. neutered (red line) female dogs.

Coefficient model estimates are reported in Table 2. With increasing dog size, an acceleration of time to malignant tumor diagnosis (*p* < 0.001) is observed. Mesocephalic dogs have a decelerated time to diagnosis, especially in comparison to brachycephalic dogs (*p* < 0.001). The interacting parameters of sex and neutering status indicate that neutering female dogs may protect against early diagnosis of malignant tumor (*p* < 0.001), as also suggested by Figure 6. However, this protective effect seems to be negligible in male dogs. In addition, these findings suggest that when only intact dogs are considered, female dogs have an acceleration of time to malignant tumor diagnosis in comparison with males.

Figure 6 shows that, roughly after the age of 3, the estimated survival function of neutered female dogs is greater than the estimated survival function of intact female dogs.

## 4. Discussion

This study evaluated the age at diagnosis for more than 14,000 tumor samples of Italian dogs submitted for histopathologic evaluation and collected at the pathology-based UNIPI ACR over a fifteen-year period (2008–2023). It represents the first aggregate survey of age at tumor diagnosis in dogs in Europe. A wide variability in age distribution was found, with significant influences of sex, neutering status, breed, size, and morphological characteristics such as cephalic index.

The overall age at tumor diagnosis was comparable to previously reported findings in other populations [23,31,32,33,34,35,37,41] and pathology-based ACRs [27,28,36,39,40], in which the highest number of tumors was reported in dogs between the ages of 9 and 11 years.

### 4.1. Tumor Behavior

A significantly earlier diagnosis of benign tumors (median 9 years) compared to malignant tumors (10 years) was found, consistent with previous studies [27,35,39,41,55]. These findings are in line with the hypothesis that cancer is the result of a complex process in which random genetic and epigenetic mutations accumulate over time. Multiple checkpoint mechanisms must be deactivated before a malignant phenotype is reached in humans [56,57]. This process also appears to occur in dogs, albeit on a shorter time scale, since the life expectancy of a dog is considerably shorter than that of a human [2].

### 4.2. Sex and Neutering Status

Comparison of age at tumor diagnosis between sexes revealed differences from the previous findings reported by Rafalko et al. in which intact males were diagnosed at a younger age compared to intact females [42]. Although univariate statistics did not reveal any significant differences between intact males and females in our sample, the multivariate model showed that intact female dogs had an accelerated time to first diagnosis of a malignant neoplasm, relative to neutered females. Intact females also had an accelerated time to first malignancy diagnosis compared to intact males. This observed discrepancy may be explained by the already known differences in demographic characteristics and tumor distribution between the Italian and American dog populations [23,28,42]. The intact/neutered ratio in the Italian canine population is clearly higher than in the United States, especially in male dogs [58,59]. The distribution of tumors in Europe and the United States may differ due to differences in neutering behavior and breed trends, and environmental factors. In addition, Rafalko et al.’s study included three separate cohorts of canine tumor patients, with lymphoma and osteosarcoma accounting for approximately half of all tumors [42]. These previous findings may not fully reflect clinical practice scenarios, particularly in Europe, where sex-specific malignancies (e.g., mammary tumors) are prevalent [23,24,25,26,28,35,38,39,41]. Thus, differences in geography and data sources (e.g., population- or pathology-based registries, insurance agencies, private laboratories) should always be taken into account when comparing results from different studies.

Despite the differences outlined above, our results on the influence of neutering status closely align with previous findings [27,28,42]. On average, neutered dogs were diagnosed with malignant tumors 7 months (males) and 6 months (females) later than their intact counterparts. The AFT model data, which takes into account multiple factors, partially corroborated the univariate statistics results. The model suggested that neutering has a significant decelerating effect on the time to diagnosis in female dogs, while it seems that its contribution in males is not as effective.

### 4.3. Size and Cephalic Index

When size and cephalic index were considered, both univariate and multivariate statistics showed an influence of these variables on the time of cancer diagnosis. An inversely proportional relationship between size and age at cancer diagnosis has been described [42], and several studies have reported that the increase in size not only reduced life expectancy, but also increased cancer mortality risk [14,60,61]. While cancer risk is largely independent of size across species, as described by Peto’s Paradox [62,63], the evolutionary model of cancer predicts that cancer risk increases with size within species. This phenomenon should also apply to dogs, due to greater cell numbers and higher cell division rates in larger individuals [5,60,61].

However, this study suggests that factors other than body size may also play a role in age at cancer diagnosis. For example, morphological features of dog breeds have been shown to have a significant impact on health outcomes in dogs, strongly influencing longevity and overall well-being [51]. In addition, the present study is the first to show that brachycephalic and dolichocephalic dogs are diagnosed with cancer at a younger age than mesocephalic breeds, after adjustment for sex, neutering status, and size. Canine brachycephaly is a human creation, with a wide range of cephalic indices in domesticated dogs, ranging from 42 to 87, compared to the wolf (*Canis lupus*), which ranges from 51 to 52 [64]. Brachycephalic breeds showed considerably higher inbreeding patterns than non-brachycephalic breeds, but lower median body weights [65]. The relationship between size and inbreeding is currently under investigation, and higher inbreeding has been reported in large dogs [60,66]. Assessing the effect of these two variables on cancer predisposition is of great interest in veterinary geriatrics [60].

### 4.4. Breed

One study evaluating the lifetime prevalence of malignant and benign tumors in a cohort of 27,541 dogs found no differences between purebred and mixed-breed dogs, but an increase in prevalence with increasing dog size [67]. Conversely, other studies reported a higher tumor incidence in purebred dogs than in mixed-breed dogs [24,25,30,35,68]. We found that purebred dogs were significantly younger than mixed breeds at the time of cancer diagnosis, with an onset ~14 months earlier, supporting the role of low genetic diversity in cancer predisposition [15,42,69]. This trend was confirmed by the specific analysis of histotypes. It is well known that different tumor types have different ages at diagnosis. The mean and median ages at diagnosis for the most common cancer groups in this study are consistent with those reported in the literature [42,47,70,71,72,73,74,75]. Our data showed that purebred dogs were significantly younger at the time of diagnosis for each of the most common malignant histotypes except for lymphoma.

The lack of conclusive results in the veterinary literature may be due to the fact that, when subjects are grouped under the umbrella of “purebred”, an extremely heterogeneous group is formed. As demonstrated by our and previous studies with a more breed-specific perspective [35,40], this type of crude comparison may obscure significant tumor predispositions, suggesting that the etiology of malignant tumors differs between breeds. We showed that the median age by the most common breeds exhibits considerable variability, with several breeds having significant early and late tumor development compared to mixed-breeds. It was shown that life expectancies and ages at death due to cancer differ by breed, too [12,60,76]. A comparison of these data is beyond the scope of the present study. However, the presence of French Bulldog, Pug, Beagle, Jack Russell Terrier, and Pinscher among the breeds with early cancer diagnosis confirmed that, while in some breeds the differences in age at cancer diagnosis may be explained by size, in other small-sized breeds the involvement of other factors is conceivable [42]. Therefore, any cancer screening plan should be tailored to the individual breed.

### 4.5. The EHA Model

The AFT models are parametric EHA models in which the covariates have a direct effect on the estimated time before the occurrence of the event of interest, rather than on the hazard itself [77]. This type of investigation is widely used in epidemiological studies in human oncology [78]. In veterinary medicine, EHA methods have been used to study life expectancy [12,66,79,80]. To the authors’ knowledge, this study is the first to use this model to predict the time of cancer diagnosis in dogs. Our model showed a clear accelerating effect of large size, brachy- and dolichocephaly, and sexual integrity in females on the time to first malignant tumor diagnosis.

We acknowledge that the creation of a competitive risk model encompassing “non-tumor”, “benign tumor”, and “malignant tumor” would have been enhanced by the availability of census population data. Since data on the at-risk population were not available, the predicted hazard rate was obtained by comparing the time to the first diagnosis of malignant tumor (event 1) with the time to the first diagnosis of benign tumor (event 0). In addition, the effect of neutering should be interpreted with caution because this variable was modeled as time-independent, as exact time at which dogs underwent neutering was not known. Future studies using data from population-based registries or with a prospective design, are warranted to confirm these findings [81].

### 4.6. Research Limitations and Strengths

A limitation of the study is that the experimental design was based on histopathologic diagnoses. Cytological diagnoses of neoplasia were omitted to ensure a higher data validity, although this may not accurately reflect the clinical scenario. Despite the selection bias, the correspondence of the topographic distribution of the tumors and the demographic features in our dataset with the results of other pathology- and population-based ACRs shows a good representativeness [19,23,24,25,28,59]. Another drawback is that the age at diagnosis is a surrogate and overestimation of the true age of tumor onset, which is often inapparent. Studies of tumor growth kinetics in dogs suggest a wide variability in tumor doubling times (i.e., the time it takes for a tumor to double in size), with a latency period of over 2 years between the carcinogen exposure and the development of a clinically detectable mass [82,83,84,85]. A non-linear trend in cancer growth has also been reported, with rapid growth at the onset of tumor development, followed by a decrease in growth rate when a clinically detectable size is reached, as described by the Gompertz curve [86]. Based on these data, it has been suggested that cancer screening in dogs should begin approximately 2 years before the median age of cancer diagnosis [42]. According to our findings, it is advisable to start cancer screening in mixed-breeds at 7–8 years of age, depending on the dog size, and before 6 years of age in early tumor developing breeds.

The strengths of this study include the largest number of subjects included in this type of analysis to date and the reliability of the diagnostic criteria. Our data are based on uniform diagnostic assays and periodic agreement studies are performed with other national and international histopathologic diagnostic centers [28,87].

An additional advantage in terms of standardization of data collection, inclusion criteria, and tumor classification is the adoption of Vet-ICD-O-canine-1 [22]. The use of common and up-to-date coding systems is an essential aspect to allow comparability between studies conducted in different geographical areas and ultimately potential comparison with human data in the perspective of comparative oncology and the *One Health* approach [20,21,22].

Finally, the use of multivariate analysis with AFT models allows for more consistent data analysis in dealing with interactions and relationships among multiple covariates. This is particularly true for the variable “age”, given its non-normal distribution and the consequent need to use non-parametric univariate statistics.

The identification of different age distributions among canine groups, as well as the observed associations between risk factors and the onset of cancer diagnosis in dogs, have important clinical implications, providing insights into the interplay between size, morphology, inbreeding, and tumor predisposition. A strong relationship between clinicians and pathologists is required to acquire significant and large datasets. Knowing that accurate data will impact epidemiological research, clinicians should be more active in providing reliable information to pathologists. This same information will then allow for the development of solid data that should help the clinician make better medical decisions and thus improve the health of our pets.

## 5. Conclusions

The present findings provide insight into the influence of sex, neutering status, morphologic and breed characteristics on the age at tumor diagnosis in dogs. Large size, dolicho- and brachycephaly, and sexual integrity in females appear to accelerate the onset of malignancy. This study confirms that a “one-size-fits-all” approach to cancer screening is not the most effective one in the canine population, and we hope it will provide relevant guidance for future investigations and facilitate the establishment of more personalized cancer screening programs in dogs.

## Figures and Tables

**Table 1:** Number of dogs and total and malignant tumor cases included in this study by sex, neutering status, breed, size, and cephalic index.

Variables			Dogs		Total Tumors		Malignant Tumors	
			n	%	n	%	n	%
Sex	Male	Intact	4984	37.8	5486	37.4	2659	30.7
		Neutered	644	4.9	689	4.7	441	5.1
		N.d.	4	<0.1	4	<0.1	4	<0.1
	Female	Intact	4718	35.8	5318	36.2	3510	40.5
		Neutered	2561	19.4	2847	19.5	1876	21.6
		N.d.	1	<0.1	1	<0.1	1	<0.1
	N.d.		277	2.1	291	2.0	177	2.0
Breed		Mixed-breed	4822	36.6	5303	36.2	3146	36.3
		Purebred	8367	63.4	9333	63.8	5522	63.7
Size ^1^		Small	2122	25.4	2351	25.2	1323	24.0
		Medium	1740	20.8	1943	20.8	1152	20.9
		Large	4209	50.3	4700	50.4	2862	51.8
Cephalic index ^1^		Brachycephalic	1415	16.9	1586	17.0	988	17.9
		Mesocephalic	4886	58.4	5458	58.5	3150	57.0
		Dolichocephalic	2051	24.5	2274	24.4	1373	24.9

^1^ Size and cephalic index data were available only for purebred subjects due to the heterogeneous phenotype within the mixed-breed group, thus these percentages refer to the total purebred subset. For sex and breed, the percentages refer to the entire dataset. N.d. = Not determined. n = number.

**Table 2:** Coefficient model estimates (of purebred dogs) with *p* values generated using the Accelerated Failure Time (AFT) model. Note that Female # Intact shows the interaction between sex and neutering status.

Covariates	Variables	CE (*p* Value) ^1^
Sex	Male Female	Ref
		−0.01 (0.667)
Neutering status	Neutered	Ref
	Intact	0.02 (0.426)
Sex # Neutering status	Female # Intact	−0.10 (<0.001)
Size	Small	Ref
	Medium	−0.06 (<0.001)
	Large	−0.12 (<0.001)
Cephalic index	Mesocephalic	Ref
	Brachycephalic	−0.15 (<0.001)
	Dolichocephalic	−0.02 (0.034)

^1^ Coefficient estimates; unit: year. Ref = Reference.

## Data Availability

The data that support the results reported in this article are openly available in the Zenodo public data repository at https://zenodo.org/doi/10.5281/zenodo.13685501 (accessed on 3 September 2024).

## Supplementary Materials

The following supporting information can be downloaded at: https://www.mdpi.com/article/10.3390/vetsci11100485/s1, Table S1: Distribution of tumor cases (sample size and percentage of the total) in the UNIPI Animal Cancer Registry (2008–2023) per tumor topography group, morphology, and according to the biological behavior; Table S2: Age at malignant tumor diagnosis (range, mean, and median) for the 156 individual breeds included in the UNIPI Animal Cancer Registry (2008–2023), along with body size (small; medium; large), and cephalic index (brachycephalic; mesocephalic; dolichocephalic) classification and number of tumors.
