# Supplementary material for: Age at Tumor Diagnosis in 14,636 Canine Cases from the Pathology-Based UNIPI Animal Cancer Registry, Italy: One Size Doesn’t Fit All
Source: Vet Sci. 2024 Oct 8;11(10):485. doi: 10.3390/vetsci11100485 (PMC11512385; doi:10.3390/vetsci11100485)
Supplement: Supplementary file 1 [file vetsci-11-00485-s001.zip › Supplementary/Table S2.pdf]

Table S2. Age at malignant tumor diagnosis (range, mean, and median) for the 156 individual breeds included in the UNIPI Animal Cancer Registry (2008-2023), along with body size (small; medium; large), and cephalic index (brachycephalic; mesocephalic; dolichocephalic) classification and number of tumors. Total tumors = benign + malignant tumors; min = youngest age at tumor diagnosis; max = oldest age at tumor diagnosis; n = number.

Breeds (n = 156)	Body Size	Cephalic Index	Total tumors (n)	Malignant tumors (n)	Age at malignant tumor diagnosis (years)			
					min	max	mean	median
Afghan Hound	Large	Dolichocephalic	5	3	8.0	9.0	8.3	8.0
Airedale Terrier	Medium	Dolichocephalic	11	4	8.6	11.0	9.9	10.0
Akita Inu	Large	Mesocephalic	19	9	5.6	12.0	9.2	9.0
Alaskan Malamute	Large	Mesocephalic	14	11	0.1	11.0	8.6	10.0
American Bulldog	Large	Brachycephalic	3	2	3.0	10.0	6.5	6.5
American Bully	Medium	Brachycephalic	4	3	2.0	4.0	2.9	2.6
American Pit Bull Terrier	Medium	Mesocephalic	199	129	0.9	14.0	9.1	9.5
American Staffordshire Terrier	Medium	Mesocephalic	83	59	1.0	12.0	8.1	8.0
Australian Cattle Dog	Medium	Mesocephalic	1	1	5.0	5.0	5.0	5.0
Australian Shepherd	Medium	Mesocephalic	25	14	5.0	12.0	8.3	8.5
Australian Terrier	Small	Mesocephalic	1					
Barsoi	Large	Dolichocephalic	1					
Basenji	Small	Mesocephalic	2					
Basset Hound	Medium	Dolichocephalic	25	6	5.6	10.0	7.7	7.9
Bavarian Mountain Hound	Medium	Mesocephalic	4	3	8.0	12.0	9.7	9.0
Beagle	Small	Mesocephalic	268	141	2.0	15.0	9.4	9.0
Beauceron	Large	Mesocephalic	3	2	10.0	10.0	10.0	10.0
Bedlington Terrier	Small	Dolichocephalic	1					
Belgian Shepherd Dog	Medium	Dolichocephalic	33	15	5.0	12.0	8.7	8.0
Bergamasco Shepherd Dog	Large	Mesocephalic	8	7	6.0	13.0	10.7	11.9
Bernese Mountain Dog	Large	Mesocephalic	76	33	2.5	12.0	7.7	8.0
Bichon Frisé	Small	Mesocephalic	15	4	4.0	11.0	8.5	9.5
Black Russian Terrier	Large	Mesocephalic	2	1	12.0	12.0	12.0	12.0
Bobtail (Old English Sheepdog)	Large	Mesocephalic	6	1	15.0	15.0	15.0	15.0
Bolognese	Small	Mesocephalic	47	27	3.0	15.6	10.3	11.0
Border Collie	Medium	Mesocephalic	70	34	2.0	16.0	9.1	9.2
Boston Terrier	Small	Brachycephalic	7	6	5.0	11.0	7.7	7.5
Bouvier de Flandres	Large	Mesocephalic	3	3	7.0	8.0	7.7	8.0
Boxer	Large	Brachycephalic	803	534	1.6	15.0	7.7	8.0
Briard	Large	Mesocephalic	22	12	5.0	13.0	9.3	8.5
Bull Terrier	Medium	Dolichocephalic	14	5	5.0	16.0	9.1	7.0
Bulldog	Medium	Brachycephalic	78	37	1.0	12.0	7.2	8.0
Bullmastiff	Large	Brachycephalic	26	19	5.0	11.0	7.6	8.0
Cairn Terrier	Small	Mesocephalic	4	4	1.6	14.0	9.7	11.5
Canarian Warren Hound	Small	Dolichocephalic	1	1	9.0	9.0	9.0	9.0
Caucasian Shepherd Dog	Large	Mesocephalic	5	4	6.0	9.0	7.8	8.0
Cavalier King Charles Spaniel	Small	Brachycephalic	41	18	3.0	14.6	8.2	8.5
Central Asia Shepherd Dog	Large	Mesocephalic	2	1	6.0	6.0	6.0	6.0
Chihuahua	Small	Brachycephalic	58	43	4.0	13.0	8.9	9.0
Chinese Crested Dog	Small	Mesocephalic	3	1	8.0	8.0	8.0	8.0
Chow Chow	Large	Mesocephalic	17	10	5.0	13.0	9.5	9.5
Cirneco dell'Etna	Medium	Dolichocephalic	3	2	11.0	13.0	12.0	12.0
Cocker Spaniel	Small	Mesocephalic	313	148	1.8	16.0	9.9	10.0
Collie	Medium	Dolichocephalic	19	10	7.0	13.0	9.8	10.0
Continental Toy Spaniel	Small	Mesocephalic	3	3	5.0	11.0	7.7	7.0
Coton de Tulear	Small	Mesocephalic	2	1	6.0	6.0	6.0	6.0
Czechoslovakian Wolfdog	Large	Dolichocephalic	17	8	7.0	11.5	9.5	9.6
Dachshund	Medium	Dolichocephalic	244	144	1.5	18.0	9.7	10.0
Dalmatian	Medium	Mesocephalic	52	27	7.0	14.0	11.4	12.0
Do Khyi (Tibetan Mastiff)	Large	Brachycephalic	4	2	8.0	14.0	11.0	11.0
Dobermann	Medium	Dolichocephalic	183	135	2.0	15.0	8.3	8.0
Dogo Argentino	Large	Mesocephalic	88	64	2.0	13.0	7.7	8.0
Dogue de Bordeaux	Large	Brachycephalic	46	21	0.7	11.0	6.0	7.0
Elo	Large	Mesocephalic	1					
English Pointer	Medium	Mesocephalic	52	34	4.0	13.0	9.0	9.0
Entlebuch Cattle Dog	Medium	Mesocephalic	1					
Epagneul Breton	Medium	Mesocephalic	246	160	4.0	18.0	10.0	10.0
Fila Brasileiro	Large	Brachycephalic	1	1	10.0	10.0	10.0	10.0

Flat Coated Retriever	Large	Mesocephalic	13	8	3.0	13.0	8.9	10.0
Fox Terrier	Small	Dolichocephalic	53	32	4.0	14.0	9.9	10.0
French Bulldog	Small	Brachycephalic	118	70	2.5	14.0	8.2	8.0
French Pointing Dog	Medium	Mesocephalic	4	4	6.0	10.0	8.5	9.0
Galgo Espanol	Large	Dolichocephalic	3	1	8.0	8.0	8.0	8.0
German Hunting Terrier	Medium	Mesocephalic	6	3	7.0	16.0	11.7	12.0
German Shepherd	Large	Dolichocephalic	899	561	1.0	20.0	9.1	9.0
German Shorthaired Pointing Dog	Medium	Mesocephalic	76	43	3.0	15.0	8.8	9.0
Golden Retriever	Large	Mesocephalic	498	276	1.0	16.0	8.7	9.0
Gos d'Atura Catala	Medium	Mesocephalic	1					
Great Dane	Large	Dolichocephalic	23	14	4.0	10.6	7.0	7.0
Greater Swiss Mountain Dog	Large	Mesocephalic	4	4	6.0	9.0	7.8	8.1
Greyhound	Large	Dolichocephalic	2					
Griffon NOS	Medium	Mesocephalic	1					
Hanover Hound	Large	Mesocephalic	1					
Hovawart	Large	Mesocephalic	8	2	9.0	10.0	9.5	9.5
Hungarian Short-Haired Pointer (Vizsla)	Medium	Mesocephalic	7	5	4.2	12.0	7.8	8.0
Irish Soft Coated Wheaten Terrier	Medium	Mesocephalic	2	1	10.0	10.0	10.0	10.0
Irish Wolfhound	Large	Dolichocephalic	1	1	4.0	4.0	4.0	4.0
Italian Cane Corso	Large	Brachycephalic	127	71	1.5	13.0	8.2	8.0
Italian Pointing Dog	Large	Dolichocephalic	23	10	3.0	14.0	9.5	10.0
Italian segugio NOS	Large	Dolichocephalic	79	49	3.0	15.0	8.2	8.0
Italian Short-Haired Segugio	Large	Dolichocephalic	27	17	2.5	12.0	7.6	8.0
Italian Sighthound	Small	Dolichocephalic	8	5	11.0	15.0	13.2	13.0
Italian Volpino	Small	Mesocephalic	48	24	4.0	15.0	10.7	10.5
Jack Russell Terrier	Small	Mesocephalic	226	132	0.1	18.5	9.0	9.0
Japanese Chin	Small	Brachycephalic	1	1	3.0	3.0	3.0	3.0
Karelian Bear Dog	Medium	Mesocephalic	1					
Kerry Blue Terrier	Medium	Dolichocephalic	1	1	7.0	7.0	7.0	7.0
Labrador Retriever	Large	Mesocephalic	809	438	0.2	15.0	9.0	9.0
Leonberger	Large	Mesocephalic	7	6	5.7	8.0	6.6	6.5
Lhasa Apso	Small	Brachycephalic	10	3	5.0	12.0	9.7	12.0
Maltese	Small	Mesocephalic	93	60	4.5	17.0	10.4	11.0
Manchester Terrier	Small	Dolichocephalic	2	1	6.0	6.0	6.0	6.0
Maremma and the Abruzzes Sheepdog	Large	Mesocephalic	103	52	3.0	14.0	9.2	9.6
Mastiff	Large	Brachycephalic	14	10	5.0	10.0	8.5	9.0
Mixed-breed	NA	NA	5,303	3,146	0.1	18.0	10.1	10.0
Münsterländer NOS	Medium	Mesocephalic	1	1	7.0	7.0	7.0	7.0
Neapolitan Mastiff	Large	Brachycephalic	2	2	5.0	7.0	6.0	6.0
Newfoundland	Large	Mesocephalic	32	16	5.0	13.0	9.4	9.5
Norfolk Terrier	Small	Mesocephalic	1					
Norwich Terrier	Small	Mesocephalic	2	2	8.0	14.0	11.0	11.0
Nova Scotia Duck Tolling Retriever	Medium	Mesocephalic	6	2	8.0	8.0	8.0	8.0
Parson Jack Russell Terrier	Small	Mesocephalic	3					
Pinscher NOS	Small	Dolichocephalic	205	133	0.1	15.0	8.5	9.0
Pekinese	Small	Brachycephalic	23	14	6.0	14.0	10.9	11.0
Petit Bleu de Gascogne (Small Blue Gascony)	Medium	Dolichocephalic	2	1	9.1	9.1	9.1	9.1
Pointing dog NOS	Medium	Mesocephalic	32	19	6.0	17.0	11.1	12.0
Polish Lowland Sheepdog	Medium	Mesocephalic	2	2	11.0	11.5	11.3	11.3
Poodle	NA	Dolichocephalic	324	174	1.3	22.0	9.9	10.0
Portuguese Sheepdog	Medium	Mesocephalic	2					
Portuguese Water Dog	Medium	Mesocephalic	1	1	10.0	10.0	10.0	10.0
Posavatz Hound	Medium	Mesocephalic	1					
Presa Canario	Large	Mesocephalic	4	4	10.0	11.0	10.8	11.0
Pug	Small	Brachycephalic	74	50	1.0	14.0	7.7	8.0
Pyrenean Mastiff	Large	Brachycephalic	2	1	8.0	8.0	8.0	8.0
Pyrenean Mountain Dog	Large	Mesocephalic	4	2	8.0	8.0	8.0	8.0
Retriever NOS	NA	NA	1					
Rhodesian Ridgeback	Large	Mesocephalic	21	16	1.0	12.0	7.4	8.0
Romagna Water Dog	Small	Mesocephalic	57	33	3.0	14.0	7.9	8.0
Rottweiler	Large	Mesocephalic	203	145	0.1	13.0	8.2	8.0
Saluki	Medium	Dolichocephalic	1	1	7.0	7.0	7.0	7.0
Samoyed	Medium	Mesocephalic	23	12	7.6	14.0	10.8	11.0
Sardinian Shepherd Dog	NA	NA	1	1	12.0	12.0	12.0	12.0
Schipperkee	Small	Mesocephalic	1	1	11.0	11.0	11.0	11.0
Schnauzer (miniature)	Small	Mesocephalic	15	9	5.0	15.0	9.7	10.0
Schnauzer (standard)	Medium	Mesocephalic	100	64	5.0	16.0	10.1	10.0

Schnauzer (giant)	Large	Mesocephalic	5	3	8.0	12.0	10.0	10.0
Schnauzer NOS	NA	NA	120	76	5.0	16.0	10.0	10.0
Schweizer Laufhund	Medium	Dolichocephalic	1	1	7.1	7.1	7.1	7.1
Scottish Terrier	Small	Dolichocephalic	6	5	7.0	10.0	8.2	7.0
Segugio Maremmano	Large	Dolichocephalic	8	5	5.0	10.0	8.2	9.0
Serbian Hound	Medium	Mesocephalic	2					
Setter	Large	Mesocephalic	531	359	1.7	17.0	9.0	9.0
Shar Pei	Medium	Mesocephalic	31	24	2.0	13.0	8.0	8.3
Shepherd NOS	NA	NA	5	4	7.0	10.0	8.3	8.0
Shetland Sheepdog	Small	Dolichocephalic	1					
Shiba Inu	Small	Mesocephalic	9	5	7.0	11.0	9.4	10.0
Shih Tzu	Small	Brachycephalic	143	79	2.9	17.0	10.1	10.0
Siberian Husky	Medium	Mesocephalic	140	71	5.0	16.0	11.2	11.0
Sighthound NOS	Large	Dolichocephalic	16	12	3.6	13.0	9.1	9.0
Skye Terrier	Small	Mesocephalic	1					
Spanish Mastiff	Large	Brachycephalic	1	1	9.0	9.0	9.0	9.0
Spinone Italiano	Large	Mesocephalic	23	15	4.0	15.0	8.8	9.0
Spitz	Small	Mesocephalic	15	10	3.0	14.0	9.7	9.5
Springer Spaniel	Medium	Mesocephalic	150	73	3.0	14.0	9.1	10.0
St. Bernard	Large	Mesocephalic	5	1	6.0	6.0	6.0	6.0
Staffordshire Bull Terrier	Small	Mesocephalic	23	11	2.5	12.0	8.6	9.0
Tatra Shepherd Dog	Large	Mesocephalic	1	1	9.0	9.0	9.0	9.0
Terrier NOS	NA	NA	8	6	5.0	9.0	7.5	8.0
Tibetan Terrier	Small	Mesocephalic	1					
Transylvanian Hound	Medium	Dolichocephalic	1	1	9.0	9.0	9.0	9.0
Weimaraner	Large	Mesocephalic	23	7	4.0	13.0	8.7	8.0
Welsh Corgi	Small	Mesocephalic	1	1	8.0	8.0	8.0	8.0
Welsh Terrier	Small	Dolichocephalic	5	1	12.0	12.0	12.0	12.0
West Highland White Terrier	Small	Mesocephalic	93	42	6.0	16.0	10.7	10.3
Whippet	Small	Dolichocephalic	23	11	1.3	14.0	9.1	9.0
White Swiss Shepherd Dog	Large	Dolichocephalic	3	3	5.0	10.0	7.7	8.0
Wire-Haired Pointing Griffon Korthals	Medium	Mesocephalic	1					
Yorkshire Terrier	Small	Mesocephalic	324	191	2.0	17.0	10.5	11.0
Yugoslavian Shepherd Dog (Sharplanina)	Large	Mesocephalic	3	1	10.0	10.0	10.0	10.0
