# Supplementary material for: Age at Tumor Diagnosis in 14,636 Canine Cases from the Pathology-Based UNIPI Animal Cancer Registry, Italy: One Size Doesn’t Fit All
Source: Vet Sci. 2024 Oct 8;11(10):485. doi: 10.3390/vetsci11100485 (PMC11512385; doi:10.3390/vetsci11100485)
Supplement: Supplementary file 1 [file vetsci-11-00485-s001.zip › Supplementary/Table S1.pdf]

Table S1. Distribution of tumor cases (sample size and percentage of the total) in the UNIPI Animal Cancer Registry (2008-2023) per tumor topography group, morphology, and according to the biological behavior. n = number.

Variables	Vet-ICD-O-Canine1 Groups	Total tumors (n)	percentage of the total (%)	Biological behaviour	
				Benign tumors (n)	Malignant tumors (n)
Topographies (20)	C44 Skin	5,056	34.5	2,405	2,651
	C50 Mamma	3,493	23.9	752	2,741
	C49 Soft tissue	1,841	12.6	1,139	702
	C60-63 Male genital organs	959	6.6	911	48
	C00-14 Lip, oral cavity, pharynx	856	5.8	412	444
	C47,70-72 Nervous system	494	3.4	79	415
	C42 Hematopioetic system	461	3.1	16	445
	C15-26 Digestive organs	316	2.2	40	276
	C51-56 Female genital organs	266	1.8	143	123
	C40-41 Bones	187	1.3	12	175
	C77 Lymph nodes	136	0.9	9	127
	C64-68 Urinary organs	136	0.9	1	135
	C30-32 Nose, ear, sinuses, larynx	93	0.6	5	88
	C73-75 Endocrine glands	89	0.6	10	79
	C69 Eye and adnexa	83	0.6	20	63
	C33-34 Respiratory system	53	0.4	2	51
	C76,80 Peritoneum and retroperitoneum	32	0.2	8	24
	C80 Unknown	29	0.2		29
	C48 Ill defined	28	0.2	3	25
	C76,80 Intrathoracic organs (excl. lung)	28	0.2	1	27
Morphologies (43)	814-838 Adenomas and adenocarcinomas	1,956	13.4	312	1,644
	893-899 Complex mixed and stromal neoplasms	1,568	10.7	364	1,204
	974 Mast cell neoplasms	1,567	10.7	34	1,533
	839-842 Adnexal and skin appendage neoplasms	1,516	10.4	854	662
	912-916 Blood vessel tumors	825	5.6	311	514
	805-808 Squamous cell neoplasms	784	5.4	448	336
	885-888 Lipomatous neoplasms	702	4.8	671	31
	859-867 Specialized gonadal neoplasms	699	4.8	645	54
	975 Neoplasms of histiocytes and accessory lymphoid cells	568	3.9	540	28
	872-879 Melanocytoma and Melanomas	546	3.7	75	471
	809-811 Basal cell neoplasms	500	3.4	459	41
	954-957 Nerve sheath tumors	492	3.4	78	414
	881-883 Fibromatous neoplasms	419	2.9	236	183
	880 Soft tissue tumors and sarcomas, NOS	357	2.4	1	356
	906-909 Germ cell neoplasms	280	1.9	275	5
	927-934 Odontogenic tumors	259	1.8	259	
	959-972 Malignant lymphomas, NOS or diffuse	221	1.5		221
	918-924 Osseous and chondromatous neoplasms	219	1.5	11	208
	850-854 Ductal and lobular neoplasms	176	1.2	56	120
	889-892 Myomatous neoplasms	167	1.1	82	85
	973 Plasma cell neoplasms	140	1.0	140	
	884 Myxomatous neoplasms	138	0.9	26	112
	844-849 Cystic, mucinous and serous neoplasms	101	0.7	71	30
	812-813 Transitional cell papillomas and carcinomas	93	0.6	1	92
	967-972 Mature B-cell lymphomas	90	0.6		90
	801-804 Epithelial neoplasms, NOS	80	0.5	1	79
	970-971 Mature T- and NK-cell lymphomas	47	0.3		47
	856-857 Complex epithelial neoplasms	44	0.3		44
	800 Neoplasms, NOS	14	0.1		14
	868-871 Paragangliomas and glomus tumors	12	0.1		12
	900-903 Fibroepithelial neoplasms	8	0.1		8
	904 Synovial-like neoplasms	8	0.1	8	
	905 Mesothelial neoplasms	7	0.0		7
	858 Thymic epithelial neoplasms	6	0.0		6
	981-983 Lymphoid leukemias	6	0.0		6
	917 Lymphatic vessel tumors	5	0.0	3	2
	855 Acinar cell neoplasms	4	0.0		4
	984-993 Myeloid leukemias	4	0.0		4
	958 Granular cell tumors	3	0.0	3	
	926 Miscellaneous bone tumors	2	0.0	2	
	911 Mesonephromas	1	0.0	1	
	935-937 Miscellaneous tumors	1	0.0	1	
	949-952 Neuroepitheliomatous neoplasms	1	0.0		1
Total		14,636		5,968	8,668
